# CT-Derived Predictors of Incisional Hernia Following Colorectal Cancer Resection: A Systematic Review

**DOI:** 10.3390/diagnostics16152345

**Published:** 2026-07-27

**Authors:** Mohamed Alfatih Hamza, Omar Abu Saadeh, Hugo C. Temperley, Niall J. O’Sullivan, Benjamin M. Mac Curtain, Mohammed Khalifa, Abdelrazig Salih, Peter Beddy, Paul H. McCormick, Jeeban Paul Das, Michael E. Kelly

**Affiliations:** 1 Department of Surgery, St. James’s Hospital, D08 NHY1 Dublin, Ireland; 2 Royal College of Surgeons in Ireland, D02 YN77 Dublin, Ireland; 3 Department of Radiology, St. James’s Hospital, D08 NHY1 Dublin, Ireland; 4 Trinity St James Cancer Institute, St. James’s Hospital, D08 NHY1 Dublin, Ireland; kellym11@tcd.ie; 5 Northwell Health, New Hyde Park, NY 11040, USA; 6 Department of Urology, Lenox Hill Hospital, New York, NY 10075, USA; 7 Department of Surgery, Limerick University Hospital, V94 F858 Limerick, Ireland; 8 Department of Surgery, Trinity College Dublin, University of Dublin, D02 PN40 Dublin, Ireland; 9 Memorial Sloan Kettering Cancer Center, New York, NY 10065, USA; 10 School of Medicine, Trinity College Dublin, D02 PN40 Dublin, Ireland

**Keywords:** incisional hernia, colorectal cancer, computed tomography, body composition, sarcopenia, visceral fat, systematic review

## Abstract

**Background/Objectives:** To systematically review computed tomography (CT)-derived body composition and morphological predictors of incisional hernia (IH) following colorectal cancer (CRC) resection. **Methods:** PubMed/MEDLINE, Embase, Scopus, Web of Science, Cochrane Library, and CINAHL were searched from inception to May 2026, supplemented by grey literature searching. Studies reporting CT-derived predictors of IH after CRC resection were eligible. Two independent reviewers screened records, extracted data, and assessed risk of bias using the Newcastle–Ottawa Scale. Given heterogeneity in CT metrics, exposure definitions, and statistical models, findings were synthesised narratively, and certainty of evidence was assessed using GRADE. **Results:** Nine retrospective cohort studies including 2392 patients met the inclusion criteria. IH incidence ranged from 4.5% to 33.6%. Visceral adiposity was the most frequently evaluated predictor, although only two studies reported standalone adjusted odds ratios for the elevated visceral fat area. Subcutaneous adiposity, sarcobesity, and novel umbilical morphological metrics, including umbilical fat, intraperitoneal thickness, and enlargement of the umbilical orifice, were independently associated with IHs in individual studies. Umbilical fat showed the largest adjusted effect estimate (hazard ratio 6.56; 95% CI 2.73–15.70). Quantitative pooling was not performed as fewer than three studies reported comparable adjusted estimates for any predictor. Certainty of evidence was low to very low across all predictor categories. **Conclusions:** Preoperative CT may provide clinically useful, but currently low-certainty, body composition data for IH risk stratification after CRC resection. Prospective multicentre validation using standardised CT protocols is required before routine clinical implementation.

## 1. Introduction

Colorectal cancer (CRC) is the third most common malignancy worldwide, with approximately 1.9 million new cases diagnosed annually and a five-year survival exceeding 65% in high-income countries [1,2]. Surgical resection remains the cornerstone of curative treatment, and minimally invasive approaches have become the standard of care at specialist centres globally [3]. As cancer survivorship improves, the long-term sequelae of CRC surgery have become more prevalent, among these being incisional hernia (IH).

Incisional hernias are among the most common postoperative complications following abdominal surgery, with reported rates varying from 6.4% to 36% after CRC resection depending on the methodology of detection, follow-up duration and frequency, and surgical approach [4,5,6,7]. Computed tomography (CT)-based surveillance, now routinely used in CRC follow-up for oncological monitoring, has revealed particularly high rates; a large Scandinavian registry study identified IHs in 25.9% of patients at 1 year postoperatively on CT review [8]. Despite their high prevalence, IHs frequently go unrecognised until symptoms develop, quality of life is impaired, or surgical repair is required, a procedure associated with significant morbidity and recurrence rates of 20–30% [9,10].

Accurate preoperative identification of patients at elevated risk of IH development would allow for targeted preventive strategies: selection of the extraction site, prophylactic mesh placement, optimisation of fascial closure technique, and enhanced postoperative counselling [11,12]. Body composition, particularly the quantity and distribution of adipose tissue and skeletal muscle, is increasingly recognised as a determinant of surgical outcomes across multiple domains [13]. Importantly, most patients undergoing CRC resection receive preoperative CT imaging for oncological staging, providing a widely available dataset from which body composition can be quantified without additional patient burden or cost. In addition, as automated analysis in medical imaging advances, CT-derived assessments of body composition will likely evolve, improving treatment and health outcomes for oncology patients, including in the detection of IHs in CRC patients [14,15].

CT-based body composition analysis enables precise quantification of the visceral fat area (VFA), the subcutaneous fat area (SFA), the skeletal muscle index (SMI), and emerging morphological metrics, including subcutaneous fat thickness (SFT), intraperitoneal thickness (IPT), and umbilical fat characterisation [16,17]. Predominantly retrospective cohort studies have investigated these CT-derived metrics as predictors of IHs following CRC resection. However, findings have been heterogeneous, and no synthesis of the evidence exists. The relationship between sarcopenia and IHs remains particularly contested, with conflicting results occurring between CRC-specific data and mixed surgical populations [18,19].

This systematic review aims to synthesise all available evidence regarding CT-derived body composition and morphological predictors of IHs following colorectal cancer resection, summarise reported effect estimates for each predictor category, and identify methodological gaps to guide future prospective research.

## 2. Materials and Methods

### 2.1. Protocol and Registration

This systematic review was conducted and reported in accordance with the Preferred Reporting Items for Systematic Reviews and Meta-Analyses (PRISMA) 2020 guidelines [20]. The protocol was prospectively registered with PROSPERO (International Prospective Register of Systematic Reviews), registration number CRD420261426802. A full review protocol was not separately prepared.

### 2.2. Research Question and PICO Framework

The research question was formulated using the PICO framework:

Population: Adult patients (aged ≥18 years) undergoing elective resection for colorectal cancer (malignant disease only, excluding benign aetiologies).

Predictor/Intervention: Preoperative CT-derived body composition or morphological metrics, including visceral fat area (VFA), subcutaneous fat area (SFA), subcutaneous fat thickness (SFT), skeletal muscle index (SMI), sarcopenia, sarcobesity, psoas muscle index, intraperitoneal thickness (IPT), umbilical fat, and muscle attenuation/density.

Comparator: Patients without the CT-derived predictor of interest (e.g., non-sarcopenic versus sarcopenic; non-obese versus viscerally obese by CT measurement).

Primary outcome: Development of incisional hernia following colorectal cancer resection, confirmed clinically or radiologically (CT-confirmed preferred).

The primary hypothesis was that preoperative CT-derived body composition metrics are independent predictors of IH development following colorectal cancer resection.

### 2.3. Search Strategy

A comprehensive literature search was conducted across six electronic databases: PubMed/MEDLINE, Embase (via Ovid), Scopus, Web of Science (Clarivate), Cochrane Library (CENTRAL), and CINAHL (EBSCOhost). Searches were conducted from database inception to May 2026 with no lower date restriction. No language restrictions were applied at the search stage; non-English studies were subsequently assessed by abstract translation where available.

The search strategy combined three concept domains: colorectal malignancy and surgical resection terms, incisional hernia and related outcome terms, and CT-derived body composition terminology, combined using the Boolean operator AND. The full search string is provided in Supplementary File S1. Searches were supplemented by the manual screening of reference lists of included studies and relevant reviews. Grey literature searching included ClinicalTrials.gov, the WHO ICTRP, and EHS/ASCRS conference abstracts, with no date restrictions.

### 2.4. Study Selection

Inclusion criteria were:

1. Adult patients (≥18 years) undergoing elective colorectal cancer resection.
2. At least one preoperative CT-derived body composition or morphological metric formally reported as a predictor of IH.
3. Incisional hernia development reported as a primary or secondary outcome.
4. Minimum follow-up ≥12 months or CT-confirmed IH diagnosis.

Exclusion criteria were:

1. Benign colorectal disease.
2. Mixed populations where a CRC-specific subgroup with CT predictor data could not be extracted.
3. Studies reporting BMI as the only body composition metric (non-CT-derived).
4. Wrong outcome: IH enlargement after established IH, parastomal hernia, or hernia repair outcomes.
5. Non-human studies, case reports, editorials, review articles, and conference abstracts without extractable data.

All identified records were uploaded to Covidence (Veritas Health Innovation, Melbourne, Australia) for systematic screening and management. Duplicate records were removed automatically. Two independent reviewers (OAS and HCT) screened titles and abstracts against the eligibility criteria. Full texts of potentially eligible studies were obtained and independently assessed. Disagreements at any stage were resolved by discussion; unresolved disagreements were adjudicated by a third senior reviewer (MEK).

### 2.5. Data Extraction

Data were extracted independently by two reviewers (OAS and HCT) using a pre-piloted standardised form. Data relating to study characteristics (year, study design, country), patient demographics (age, sex, BMI, tumour site, surgical approach), CT metrics reported (type, anatomical level, cut-off values, measurement software), IH diagnosis method, follow-up duration, and outcome data (effect estimates with 95% confidence intervals, covariates adjusted for in multivariable analysis) were extracted. Discrepancies were resolved by a consensus, with MEK adjudication where required.

### 2.6. Risk of Bias Assessment

Risk of bias in observational cohort studies was assessed using the Newcastle–Ottawa Scale (NOS) [21]. The NOS evaluates three domains, including selection of study groups (four stars maximum), comparability of groups (two stars maximum), and ascertainment of the outcome of interest (three stars maximum), yielding a maximum score of nine stars. Studies scoring ≥7 stars were considered low risk of bias; scores of 5–6 were considered moderate risk; and scores ≤ 4 were considered high risk. Two reviewers independently assigned NOS scores; discrepancies were resolved by a consensus.

The certainty of evidence for each predictor category was assessed using the GRADE (Grading of Recommendations Assessment, Development, and Evaluation) framework. As all included studies were retrospective cohort studies, certainty began at “low” and was rated down for risk of bias, inconsistency, indirectness, and imprecision, in line with GRADE guidance for observational evidence. Ratings were assigned independently by two reviewers (OAS, HCT) with disagreements resolved by discussion.

### 2.7. Data Synthesis

Quantitative synthesis was planned where three or more studies reported directly comparable CT-derived predictors with extractable adjusted effect estimates for the same predictor and outcome. Effect estimates were considered suitable for quantitative synthesis only, where the same CT-derived metric was assessed using a broadly similar exposure definition and reported with corresponding 95% confidence intervals. Where studies reported hazard ratios from Cox proportional hazards models rather than odds ratios from logistic regression, both were retained as reported and interpreted as broadly comparable measures of relative effect, given the low cumulative incidence of incisional hernia across included studies; no formal conversion between measures was performed.

Where quantitative pooling was not appropriate because of heterogeneity in CT measurement protocols, predictor definitions, cut-off values, statistical models, effect measures, or adjustment strategies, findings were synthesised narratively. Studies were grouped according to CT predictor category: visceral adiposity, subcutaneous adiposity, sarcopenia or sarcobesity, and umbilical morphological metrics. Extracted effect estimates, adjustment status, CT measurement level, and diagnostic method for IHs were summarised in structured tables. A formal assessment of reporting bias was not performed, as fewer than ten studies contributed to any single predictor category.

## 3. Results

### 3.1. Study Selection

**Figure 1:**
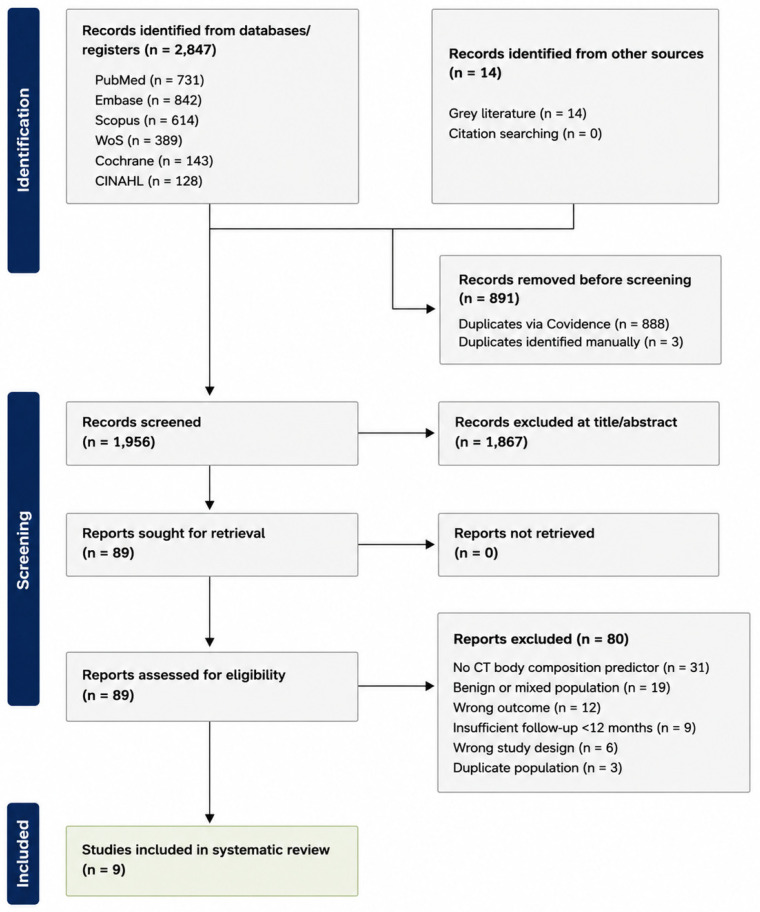
PRISMA 2020 flow diagram.

The systematic search yielded 2847 records. After removal of 891 duplicates and exclusion of 1867 records at title/abstract screening, 89 full texts were assessed, of which 80 were excluded (full breakdown in Figure 1). Nine studies met all inclusion criteria.

### 3.2. Characteristics of Included Studies

Nine studies enrolling a total of 2392 patients met the inclusion criteria (Table 1). Eight were single-centre retrospective cohort studies, and one was a multicentre retrospective cohort. Eight studies were conducted in Japan [22,23,24,25,26,27,28,29] and one in the United States [30]. Publication years ranged from 2015 to 2026. Study sample sizes ranged from 135 to 626 patients. Mean/median follow-up ranged from 17.4 to 54 months. The crude aggregate IH incidence across all studies was 13.2% (range 4.5–33.6%), with the variation likely reflecting differences in follow-up duration, IH detection method, and surgical approach; studies using CT surveillance identified higher rates than those using clinical examination alone.

Seven studies assessed visceral adiposity, including six reporting VFA and one reporting visceral fat volume. Subcutaneous adiposity was assessed in five studies, including SFA in five studies and SFT in two studies. Additional CT-derived predictors included sarcobesity or SMI (*n* = 1), umbilical fat (*n* = 1), intraperitoneal thickness with enlargement of the umbilical orifice (*n* = 1), and peritoneal protrusion at the umbilical site (*n* = 1).

### 3.3. Risk of Bias Assessment

The results of the Newcastle–Ottawa Scale risk of bias assessment are presented in Supplementary Table S1. Seven studies were rated as low risk of bias (NOS ≥ 7), and two were rated as moderate risk (NOS 5–6). No studies were considered high risk. The most prevalent sources of bias were retrospective design, present in all nine studies; single-centre enrolment, present in eight studies; and variability in IH diagnosis methodology. Three studies relied partially on clinical examination rather than systematic CT review, potentially underestimating IH incidence. Comparability was generally strong across studies, as all nine performed multivariable analysis adjusting for relevant clinical or operative confounders. Certainty of evidence for each predictor category, assessed using GRADE, is presented in Supplementary Table S3. Risk of bias due to missing results was not formally assessed for any predictor category, given the small number of contributing studies.

### 3.4. Visceral Adiposity as a Predictor of Incisional Hernia

Visceral adiposity was evaluated in several included studies, although the specific CT metric, exposure definition, and statistical model varied. Yamamoto et al. reported that VFA ≥ 110 cm^2^ was independently associated with IHs after laparoscopic CRC surgery (OR 8.45, 95% CI 2.09–34.20). Fukuoka et al. similarly identified VFA ≥ 100 cm^2^ as an independent predictor of IHs after laparoscopic colorectal surgery (OR 2.74, 95% CI 1.08–6.96). Aquina et al. reported visceral fat volume, rather than VFA, as an independent predictor using Cox regression (HR 2.04, 95% CI 1.07–3.91).

Other studies supported an association between visceral adiposity and IHs but did not provide directly comparable standalone adjusted VFA estimates. Takano et al. found visceral obesity to be associated with IHs on univariate analysis, but sarcobesity and wound infection were the independent predictors in multivariable analysis; in subgroup analysis, visceral obesity had the strongest association among sarcopenic patients. Hiraki et al. found high VFA to be associated with IHs on univariate analysis, but VFA was not retained as an independent predictor after multivariable adjustment. Sekiguchi et al. found VFA to be higher in patients who developed IHs, but only SFA remained independently predictive in multivariable analysis.

Given the heterogeneity in CT metric, cut-off value, effect measure, and adjustment model, quantitative pooling of visceral adiposity was not performed.

### 3.5. Subcutaneous Fat Metrics as Predictors

Subcutaneous adiposity was reported in five studies. Yamada et al. identified preoperative SFA as an independent predictor of IHs in a mixed open and laparoscopic CRC cohort (HR 1.012 per 1 cm^2^ increase, 95% CI 1.003–1.021, *p* = 0.005). Yamamoto et al. and Hiraki et al. also reported significant univariate associations between elevated SFA and IHs, although SFA was not consistently retained as an independent predictor after adjustment. Sekiguchi et al. found that SFA ≥ 167.8 cm^2^ was independently associated with IHs after laparoscopic CRC surgery (OR 7.73, 95% CI 1.31–45.8, *p* = 0.02).

Subcutaneous fat thickness (SFT) was evaluated as a simpler CT-derived surrogate for SFA. Tanaka H et al. identified SFT ≥ 18 mm as independently predictive of umbilical IHs, while Sekiguchi et al. showed that SFT correlated strongly with SFA and demonstrated discriminatory ability for IH prediction (AUC 0.737), outperforming BMI. Quantitative pooling of subcutaneous fat metrics was not performed because the studies differed in measurement units, cut-off values, statistical models, and reported effect measures.

### 3.6. Sarcopenia and Sarcobesity as Predictors

Skeletal muscle index (SMI) and sarcobesity were formally investigated in one CRC-specific study. Takano et al. defined sarcobesity as the coexistence of sarcopenia and visceral obesity and found that sarcobesity, together with wound infection, was independently associated with IHs after laparoscopic CRC surgery. In subgroup analysis, visceral obesity had the strongest association with IHs among sarcopenic patients (OR 13.1, 95% CI 4.51–37.8). These findings suggest that the combination of reduced skeletal muscle mass and increased visceral adiposity may confer greater abdominal wall vulnerability than either body composition abnormality alone.

### 3.7. Novel Umbilical CT Morphological Metrics

Three recent studies identified novel umbilical-level CT metrics as predictors of IHs. Tanaka H et al. demonstrated that subcutaneous fat thickness (SFT; OR 2.81, 95% CI 1.03–7.72), intraperitoneal thickness (IPT; OR 6.36, 95% CI 1.31–30.92), and enlargement of the umbilical orifice (EUO; OR 4.89, 95% CI 1.90–12.59) were independently predictive of umbilical IHs after laparoscopic CRC resection in multivariable analysis. Hiraki et al. identified lengthy protrusion of the peritoneum at the umbilical site as an independent predictor of IHs. Katayama et al. introduced the concept of “umbilical fat,” defined as preperitoneal fat tissue extending beyond the linea alba at the umbilical level without a detectable fascial defect or hernia sac, as a binary CT variable. Umbilical fat yielded the numerically largest reported adjusted effect estimate among included studies (HR 6.56, 95% CI 2.73–15.70) and was retained in multivariable Cox proportional hazards analysis.

These umbilical morphological metrics are clinically appealing because they can be assessed from standard preoperative staging CT without dedicated body composition software, requiring only visual identification or simple linear measurement at the umbilical level (Table 2).

## 4. Discussion

Across nine retrospective cohort studies enrolling 2392 patients, preoperative CT-derived body composition and morphological metrics were repeatedly associated with the development of incisional hernia following colorectal cancer resection, although heterogeneity in CT metrics and adjustment strategies precluded quantitative pooling.

Visceral adiposity was the most frequently evaluated predictor category, although only two studies (Yamamoto et al., Fukuoka et al.) reported standalone adjusted VFA estimates, with Aquina et al. reporting a related but distinct volumetric measure. Several plausible mechanisms may explain this association; visceral obesity increases intra-abdominal pressure, elevates mechanical stress at the fascial closure site, and may contribute to a pro-inflammatory environment that impairs collagen synthesis and wound healing [31], consistent with CT-based evidence that recurrent incisional hernia is associated with greater visceral and subcutaneous adiposity than primary hernia [32].

Subcutaneous adiposity also emerged as a clinically relevant predictor category. Yamada et al. identified SFA as an independent predictor of IHs in a large mixed open and laparoscopic cohort, while Sekiguchi et al. found SFA ≥ 167.8 cm^2^ to be independently associated with IHs after laparoscopic CRC surgery. SFT may be particularly practical because it can be measured using a simple linear CT measurement without specialist body composition software. Together, these findings suggest that subcutaneous adiposity may contribute to IH risk through local wound tension and technical difficulty at the extraction or incision site, complementing the intra-abdominal mechanical effects of visceral adiposity.

Sarcobesity, the coexistence of sarcopenia and visceral obesity, was the strongest body composition predictor identified (Takano et al., subgroup OR 13.1, 95% CI 4.51–37.8), supporting the hypothesis that reduced muscle tensile strength compounds the mechanical load of visceral adiposity on the abdominal wall [33]. The contrasting negative finding from van Rooijen et al. [18] may reflect population differences, including mixed benign and malignant cohorts, rather than a true absence of association in CRC patients.

The emergence of umbilical-specific morphological metrics represents the most clinically novel finding of this review. Tanaka H et al. [24], Katayama et al. [28], and Hiraki et al. [26] each identified independent umbilical-level predictors of IHs, including SFT, IPT, umbilical orifice enlargement, umbilical fat, and peritoneal protrusion, with umbilical fat showing the largest adjusted effect estimate reported across included studies. These metrics are clinically appealing as they require no specialist software and are assessable visually or by simple linear measurement on any PACS workstation.

Since preoperative staging CT is routinely performed in this population, body composition and abdominal wall morphology assessment could be incorporated into preoperative risk stratification with minimal additional imaging burden [6,34,35]. In addition, as the role of AI in radiology evolves and rapidly transforms medical imaging analysis, more effective and efficient quantification of CT-derived biomarkers will become possible, including triaging patients at increased risk of IHs based on their routine surveillance CT imaging [36,37].

Incisional hernias are multifactorial, and CT-derived body composition is only one contributor. Incision type and length, fascial closure technique, and postoperative wound infection are established determinants. In the STITCH trial, small-bite closure reduced one-year IHs from 21% to 13% (adjusted OR 0.52, 95% CI 0.31–0.87) [35], a randomised trial of transverse versus midline specimen extraction reported rates of 2% and 8% at one year [6], and European Hernia Society guidance favours continuous closure with slowly absorbable monofilament suture [11]. In the largest prospective study to place these variables within a single multivariable model, Itatsu et al. (*n* = 3927) reported hazard ratios of 1.74 (95% CI 1.28–2.38) for a midline incision, 1.68 (95% CI 1.24–2.28) for incisional surgical site infection, and 1.18 (95% CI 1.03–1.35) per 1 cm increase in subcutaneous tissue thickness [38]. The CT-derived estimates in the present review, adjusted odds ratios of 2.74 to 8.45 for elevated VFA, 7.73 for elevated SFA, and a hazard ratio of 6.56 for umbilical fat, are therefore of at least comparable magnitude, although they are taken from smaller single-centre cohorts with wide confidence intervals and are likely inflated. Formal ranking is not possible, as no included study reported suture material, bite size, or suture-length-to-wound-length ratio. Two studies did, however, enter a CT-derived metric and a conventional operative factor into the same model. Yamada et al. (SFA and open surgery) and Takano et al. (sarcobesity and wound infection) remained independently predictive, suggesting that the two act through partly distinct pathways and are complementary rather than competing. Since incision, closure, and infection prevention are modifiable at operation whereas body composition is fixed preoperatively, the value of CT lies in identifying the patients in whom attention to those modifiable factors matters most.

CT-derived metrics were the pre-specified exposure of this review; patient and clinicopathological characteristics were examined only as reported covariates rather than as exposures in their own right and were not pooled. All nine studies adjusted for a combination of patient, tumour, and operative variables, and several conventional factors retained independent predictive value, including older age and lymph node metastasis [26], age and open surgery [29], and wound infection [25]. Elevated BMI was frequently significant on univariate analysis but was superseded by CT-derived adiposity measures where the two were compared directly [27,30]. A comprehensive risk model will therefore need to combine CT-derived body composition with patient, tumour, and operative variables rather than treat them as alternatives.

Strengths include comprehensive multi-database searching, strict malignant-disease-only inclusion criteria, dual-reviewer extraction, and the first structured synthesis of this evidence base by predictor category.

Limitations primarily reflect the underlying evidence base. All studies were retrospective, and eight were single-centre cohorts, introducing selection bias and limiting generalisability. The predominance of Japanese cohorts may reduce applicability to Western populations with different body composition profiles, surgical practices, and CRC surveillance pathways. Operative technical variables known to influence IHs, including incision length, closure technique, and suture material, were inconsistently reported and rarely adjusted for, so residual confounding by surgical technique cannot be excluded. Publication bias cannot be excluded, given the small evidence base and potential underreporting of negative CT predictor studies.

## 5. Conclusions

Preoperative CT may provide clinically useful body composition and morphological data to help predict incisional hernia after colorectal cancer resection. Visceral adiposity, subcutaneous adiposity, sarcobesity, and umbilical morphological features were repeatedly associated with IHs, though heterogeneity precluded quantitative pooling. These findings support a potential role for CT-derived risk stratification in preoperative counselling, although prospective multicentre validation using standardised CT protocols is required before clinical implementation.

## Figures and Tables

**Table 1:** Characteristics of included studies.

Author (Year)	Country	*n*	IH Cases (%)	Approach	CT Metric(s)	CT Level/Method	IH Diagnosis	Follow-up (mo)	Key Predictor Finding
Aquina 2015 [30]	USA	193	41 (21.2%)	Open + Lap	Visceral fat volume, SFA, total fat	Volumetric CT; umbilical region	Clinical documentation and/or CT	Mean 25	Visceral fat volume: HR 2.04 (*p* < 0.05)
Yamada 2016 [29]	Japan	626	40 (7.3% at 5y)	Open + Lap	SFA, VFA	Umbilical level	Clinical + imaging	Median 54	SFA: HR 1.012 per cm^2^, 95% CI 1.003–1.021; open surgery: HR 4.410, 95% CI 1.018–19.095
Yamamoto 2018 [22]	Japan	212	18 (8.5%)	Laparoscopic	VFA, SFA	Umbilical level	CT (surveillance)	Mean 36	VFA ≥ 110 cm^2^: adjusted OR 8.45, 95% CI 2.09–34.20
Fukuoka 2021 [23]	Japan	423	36 (8.5%)	Laparoscopic	VFA ≥ 100 cm^2^	Umbilical level	CT (surveillance)	Mean 48	VFA ≥ 100 cm^2^: adjusted OR 2.74, 95% CI 1.08–6.96
Tanaka 2022 [24]	Japan	135	28 (20.7%)	Laparoscopic	SFT, IPT, umbilical orifice enlargement	Umbilical level	CT + clinical exam	NR	SFT, IPT, EUO: independent predictors (multivariable)
Takano 2023 [25]	Japan	262	44 (16.8%)	Laparoscopic	SMI (sarcopenia), VFA (visceral obesity), sarcobesity	L3 and umbilical level	Clinical + CT	Median 42	Sarcobesity and wound infection: independent predictors; visceral obesity in sarcopenic subgroup: OR 13.1, 95% CI 4.51–37.8
Hiraki 2024 [26]	Japan	202	52 (25.7%)	Laparoscopic	SFA, VFA, skeletal muscle area, rectus distance, peritoneal protrusion	Umbilical level	CT (surveillance)	Mean 36	Age, lymph node metastasis, and peritoneal protrusion: independent predictors; SFA/VFA significant on univariate analysis only
Sekiguchi 2025 [27]	Japan	199	9 (4.5%)	Laparoscopic	SFA, SFT, VFA	Umbilical level	CT (surveillance)	NR	SFA ≥ 167.8 cm^2^: adjusted OR 7.73, 95% CI 1.31–45.8; SFT AUC 0.737
Katayama 2026 [28]	Japan	140	47 (33.6%)	Laparoscopic	Umbilical fat (binary on CT)	Umbilical level; preperitoneal fat beyond linea alba	CT (surveillance)	Mean 17.4	Umbilical fat: HR 6.56 (95% CI 2.73–15.70, *p* < 0.01)

Abbreviations: SFA, subcutaneous fat area; VFA, visceral fat area; SFT, subcutaneous fat thickness; IPT, intraperitoneal thickness; SMI, skeletal muscle index; EUO, enlargement of umbilical orifice; IH, incisional hernia; mo, months; NR, not reported; Lap, laparoscopic; OR, odds ratio; HR, hazard ratio; AUC, area under receiver operating characteristic curve.

**Table 2:** Summary of CT-derived body composition and morphological predictors of incisional hernia reported in included studies.

CT Metric	Studies (*n*)	Total Patients	Key Reported Association	Overall Interpretation
Visceral adiposity (VFA or visceral fat volume)	7	2117	Standalone adjusted VFA associations reported by Yamamoto and Fukuoka; visceral fat volume independently associated in Aquina	Frequently associated with IHs, but heterogeneous definitions and models precluded pooling
Subcutaneous adiposity	5	1432	SFA independently associated in Yamada and Sekiguchi; SFT independently associated in Tanaka H and proposed as a surrogate in Sekiguchi	Consistent supportive evidence; practical measurement possible on routine CT
Umbilical fat	1	140	Umbilical fat independently associated with IHs in Katayama (HR 6.56, 95% CI 2.73–15.70)	Strong emerging predictor requiring external validation
Intraperitoneal thickness	1	135	IPT independently associated with umbilical IHs in Tanaka H	Simple morphological CT marker; single-study evidence
Peritoneal protrusion at umbilical site	1	202	Peritoneal protrusion independently associated with IHs in Hiraki	Morphological predictor beyond conventional adiposity measures
Umbilical orifice enlargement	1	135	EUO independently associated with umbilical IHs in Tanaka H	Simple morphological CT marker; single-study evidence
Sarcobesity/skeletal muscle depletion	1	262	Sarcobesity independently associated with IHs in Takano; visceral obesity showed strongest subgroup association among sarcopenic patients	Promising body composition phenotype, but currently supported by a single CRC-specific study

CT, computed tomography; EUO, enlargement of umbilical orifice; HR, hazard ratio; IH, incisional hernia; IPT, intraperitoneal thickness; SFA, subcutaneous fat area; SFT, subcutaneous fat thickness; VFA, visceral fat area.

## Data Availability

No new data were created or analysed in this study. Data sharing is not applicable to this article.

## Supplementary Materials

The following supporting information can be downloaded at: https://www.mdpi.com/article/10.3390/diagnostics16152345/s1. Supplementary File S1: Full PubMed search strategy; Table S1: Newcastle–Ottawa Scale risk of bias assessment; Table S2: Risk-of-bias domain-level written justifications. Table S3: GRADE certainty of evidence for CT-derived predictors of incisional hernia.
