# Supplementary material for: CT-Derived Predictors of Incisional Hernia Following Colorectal Cancer Resection: A Systematic Review
Source: Diagnostics (Basel). 2026 Jul 27;16(15):2345. doi: 10.3390/diagnostics16152345 (PMC13464622; doi:10.3390/diagnostics16152345)
Supplement: Supplementary file 1 [file diagnostics-16-02345-s001.zip › diagnostics-4437432-supplementary.pdf]

Supplementary File S1: Full PubMed Search Strategy

Search conducted in PubMed/MEDLINE via NCBI interface May 2026:

#1: ("colorectal neoplasms"[MeSH Terms] OR "colorectal cancer"[tiab] OR "colorectal carcinoma"[tiab] OR "colorectal adenocarcinoma"[tiab] OR "colon cancer"[tiab] OR "rectal cancer"[tiab] OR "sigmoid cancer"[tiab] OR "colonic neoplasms"[MeSH Terms] OR "rectal neoplasms"[MeSH Terms])
#2: ("colectomy"[MeSH Terms] OR "colectomy"[tiab] OR "colorectal resection"[tiab] OR "colorectal surgery"[tiab] OR "hemicolectomy"[tiab] OR "anterior resection"[tiab] OR "low anterior resection"[tiab] OR "abdominoperineal resection"[tiab] OR "proctectomy"[tiab] OR "proctocolectomy"[tiab] OR "laparoscopic colectomy"[tiab] OR "robotic colectomy"[tiab])
#3: #1 AND #2
#4: ("incisional hernia"[MeSH Terms] OR "incisional hernia"[tiab] OR "trocar site hernia"[tiab] OR "port site hernia"[tiab] OR "wound hernia"[tiab] OR "extraction site hernia"[tiab] OR "fascial defect"[tiab])
#5: #3 AND #4
#6: ("tomography, x-ray computed"[MeSH Terms] OR "computed tomography"[tiab] OR "CT scan"[tiab] OR "CT imaging"[tiab])
#7: ("sarcopenia"[MeSH Terms] OR "sarcopenia"[tiab] OR "myopenia"[tiab] OR "body composition"[tiab] OR "skeletal muscle index"[tiab] OR "skeletal muscle area"[tiab] OR "psoas muscle"[tiab] OR "abdominal wall thickness"[tiab] OR "linea alba"[tiab] OR "umbilical fat"[tiab] OR "visceral fat"[tiab] OR "visceral adipose tissue"[tiab] OR "subcutaneous fat"[tiab] OR "subcutaneous adipose tissue"[tiab] OR "intraperitoneal thickness"[tiab] OR "subcutaneous fat thickness"[tiab] OR "muscle attenuation"[tiab] OR "muscle density"[tiab] OR "sarcobesity"[tiab] OR "adiposity"[tiab])
#8: #5 AND #6 AND #7

Filters applied: None (no date limit, no language filter at search stage). Human studies filter applied post-search.

Table S1: Newcastle-Ottawa Scale risk of bias assessment

*NOS domains: Selection, (1) representativeness of exposed cohort; (2) selection of non-exposed cohort; (3) ascertainment of exposure; (4) outcome not present at start. Comparability, (1,2) comparability on most important factors. Outcome, (1) assessment; (2) adequacy of follow-up. * = one star; ** = two stars (maximum for comparability).*

Study	Selection 1	Selection 2	Selection 3	Selection 4	Comparability 1	Comparability 2	Outcome 1	Outcome 2	Total
Aquina 2015	*	*	*	*	**		*	*	8/9
Yamada 2016	*	*	*	*	**		*	*	8/9
Yamamoto 2018	*	*	*		**		*	*	7/9
Fukuoka 2021	*	*	*	*	**		*	*	8/9
Tanaka H 2022	*	*			*		*	*	5/9
Takano 2023	*	*	*		**		*	*	7/9
Hiraki 2024	*	*	*	*	**		*	*	8/9
Sekiguchi 2025	*	*	*		**		*		6/9

Katayama 2026	*	*	*	*	**		*	*	8/9

Table S2: Risk-of-bias domain-level written justifications

Study	Selection	Comparability	Outcome
**Aquina 2015** (8/9)	Consecutively enrolled, representative single-centre CRC cohort; comparison group drawn from the same source population; exposure (visceral fat volume) ascertained from CT performed for staging rather than self-report; No baseline IH was reported; however, retrospective ascertainment leaves some residual risk of baseline misclassification	Multivariable Cox model adjusted for the two most important confounders (inguinal hernia history, extraction site)	IH ascertained via combined chart documentation and CT; follow-up (mean 25 months) adequate in length and completeness
**Yamada 2016** (8/9)	Large consecutive single-centre cohort; adequate comparison group, exposure, and baseline-outcome ascertainment	Cox model adjusted for age and surgical approach	CT-confirmed outcome; median follow-up 54 months, long and complete
**Yamamoto 2018** (7/9)	Selection-4 star withheld: the study does not explicitly confirm exclusion of pre-existing umbilical defects at baseline, leaving some risk of outcome misclassification	Multivariable logistic model adjusting for sex and VFA	CT-surveillance-confirmed outcome; adequate follow-up
**Fukuoka 2021** (8/9)	Multicentre consecutive cohort with explicit baseline exclusion of pre-existing umbilical hernia	Adjusted for umbilical hernia history and VFA	CT-confirmed; adequate multicentre follow-up
**Tanaka H 2022** (5/9)	Selection-3 and Selection-4 withheld: exposure (EUO) ascertainment partly relies on a study-specific, not externally validated, CT definition, and baseline exclusion of pre-existing umbilical pathology is not explicitly stated	Only 1 of 2 comparability stars: multivariable model adjusts for the principal confounders but the comparison groups are not matched on hernia-relevant factors	CT- and clinical-exam-confirmed outcome; follow-up duration not separately reported, introducing uncertainty about completeness
**Takano 2023** (7/9)	Selection-4 star withheld: pre-existing umbilical/abdominal wall defects at baseline not explicitly excluded	Adjusted for wound infection and sarcobesity	CT- and clinical-confirmed; median follow-up 42 months, adequate
**Hiraki 2024** (8/9)	Single-centre but explicit baseline exclusion criteria and consecutive enrolment	Adjusted for age, lymph node metastasis, and peritoneal protrusion	CT-surveillance outcome; mean 36-month follow-up, adequate
**Sekiguchi 2025** (6/9)	Selection-4 star withheld: baseline umbilical/hernia status not explicitly excluded	Adjusted for BMI and VFA in the same model	Outcome-2 (adequacy of follow-up) star withheld: follow-up duration not stated for the primary cohort, and the validation cohort's shorter, less-defined follow-up compounds this uncertainty

**Katayama 2026** (8/9)	Consecutive cohort with explicit exclusion of prior abdominal surgery at the umbilical site	Adjusted for suturing technique and operative time	CT-surveillance outcome; adequate follow-up reported

Table S3: GRADE certainty of evidence for CT-derived predictors of incisional hernia

Predictor category	Studies (patients)	Certainty	Rationale
Visceral adiposity (VFA/volume)	7 (2,117)	⊕◯◯◯ Very low	Downgraded for serious inconsistency (adjusted effect sizes ranging 2.04–8.45 across differing cut-offs of 100 cm^2 and 110 cm^2, and a distinct volumetric metric in Aquina) and serious imprecision (Yamamoto's CI spans 2.09–34.20)
Subcutaneous adiposity (SFA/SFT)	5 (1,432)	⊕⊕◯◯ Low	Downgraded for indirectness, as studies reported effects on different scales (per-cm^2 HR in Yamada vs. threshold-based OR in Sekiguchi) and for imprecision in smaller cohorts; direction of association was reasonably consistent
Sarcobesity / SMI	1 (262)	⊕◯◯◯ Very low	Single-study evidence; consistency cannot be assessed, and the subgroup estimate (OR 13.1) carries a wide CI consistent with overestimation in a small stratum
Umbilical fat	1 (140)	⊕◯◯◯ Very low	Single-study, novel metric without external validation, despite the large point estimate
Intraperitoneal thickness	1 (135)	⊕◯◯◯ Very low	Single-study, novel metric without external validation
Enlargement of umbilical orifice	1 (135)	⊕◯◯◯ Very low	Single-study, novel metric without external validation
Peritoneal protrusion at umbilical site	1 (202)	⊕◯◯◯ Very low	Single-study, novel metric without external validation
